# Latitudinal-Related Variation in Wintering Population Trends of Greylag Geese (*Anser Anser*) along the Atlantic Flyway: A Response to Climate Change?

**DOI:** 10.1371/journal.pone.0140181

**Published:** 2015-10-14

**Authors:** Cristina Ramo, Juan A. Amat, Leif Nilsson, Vincent Schricke, Mariano Rodríguez-Alonso, Enrique Gómez-Crespo, Fernando Jubete, Juan G. Navedo, José A. Masero, Jesús Palacios, Mathieu Boos, Andy J. Green

**Affiliations:** 1 Wetland Ecology Department, Estación Biológica de Doñana (EBD-CSIC), Sevilla, Spain; 2 Department of Biology, Lund University, Lund, Sweden; 3 Office National de la Chasse et de la Faune Sauvage, Nantes, France; 4 Servicio Territorial de Medio Ambiente de Zamora, Junta de Castilla León, Zamora, Spain; 5 Sección de Espacios Naturales y Especies Protegidas, Consejería de Fomento y Medio Ambiente, Junta de Castilla y León, Palencia, Spain; 6 Avespalencia.org, Palencia, Spain; 7 Instituto de Ciencias Marinas y Limnológicas, Universidad Austral de Chile, Valdivia, Chile; 8 Grupo de Biología de la Conservación, Universidad de Extremadura, Badajoz, Spain; 9 Research Agency in Applied Ecology, Naturaconst@, Wilshausen, France; Università degli Studi di Milano-Bicocca, ITALY

## Abstract

The unusually high quality of census data for large waterbirds in Europe facilitates the study of how population change varies across a broad geographical range and relates to global change. The wintering population of the greylag goose *Anser anser* in the Atlantic flyway spanning between Sweden and Spain has increased from 120 000 to 610 000 individuals over the past three decades, and expanded its wintering range northwards. Although population sizes recorded in January have increased in all seven countries in the wintering range, we found a pronounced northwards latitudinal effect in which the rate of increase is higher at greater latitudes, causing a constant shift in the centre of gravity for the spatial distribution of wintering geese. Local winter temperatures have a strong influence on goose numbers but in a manner that is also dependent on latitude, with the partial effect of temperature (while controlling for the increasing population trend between years) being negative at the south end and positive at the north end of the flyway. Contrary to assumptions in the literature, the expansion of crops exploited by greylag geese has made little contribution to the increases in population size. Only in one case (expansion of winter cereals in Denmark) did we find evidence of an effect of changing land use. The expanding and shifting greylag population is likely to have increasing impacts on habitats in northern Europe during the course of this century.

## Introduction

Global warming is unequivocal: the mean surface temperature of the Earth has increased about 0.85°C since 1880, when long-term recording started at multiple sites [1], and there is high confidence that the average annual temperatures in the Northern Hemisphere over the period 1983–2012 have been the warmest for the last 800 years [1]. There is ample evidence of the ecological impacts that this rise in temperature has had on range shifts to keep up with climate change [2–4]. However, for taxa with a widespread distribution the effects on changes in abundance in different parts of their range are much less clear, because reliable census data are rarely available from many parts of this range. The quality of census data for large, conspicuous waterbirds such as geese are often particularly good, and especially in Europe where a high human density and strong ornithological tradition can facilitate intensive monitoring over large areas.

In the Northern hemisphere, migratory birds usually fly long distances between breeding and wintering grounds, spending the winter at lower latitudes, thus taking advantage of seasonal changes in food availability and day length [5]. At higher latitudes, milder winter conditions due to climate warming may allow birds to remain near to the breeding grounds during winter. A pattern of colonization from lower to higher latitudes so as to occupy the newly available habitats may be expected. The main potential advantages of wintering near the breeding grounds are to avoid the mortality associated with migration, to arrive earliest and in better condition at the breeding grounds, and to occupy the highest quality habitat, enhancing reproductive success [6–8]. On the other hand, the main disadvantage is a high thermoregulatory cost as a consequence of more unfavorable winter conditions and sudden changes in availability of resources (e.g. due to snow fall) [9–10].

In the case of waterbirds, changes in migratory phenology have been reported in relation to predation risk [11], or climate change, the latter including both the advancement of spring migration [e.g. 12–16] and delay of autumn migration [17]. Changes in the distribution of wintering populations have also been recorded, usually representing a northward shift of geographical ranges [e.g. 18, 15, but see 19–20]. These changes are thought to be mainly related to climate change, especially rising temperatures [e.g. 5, 21–23]. However, changes in land-use have also played an important role and some migratory waterbirds have responded positively to the intensification of agriculture or the creation of refuges [e.g. 24–26].

Wintering waterfowl populations have been monitored for decades across Europe, producing long-term datasets on bird numbers and distribution (http://www.wetlands.org). Among these species, one of the best studied is the European greylag goose (*Anser anser*), whose populations breeding in Norway, southern Sweden, Denmark, northern Germany, the Netherlands and Belgium use the Atlantic migratory flyway [27]. Because of the broad wintering range of this flyway population across countries where all major wetlands have been counted for decades, it provides a unique opportunity to relate changes in distribution to population trends across the range, and to different aspects of global change.

For most of the 20th century, the majority of greylags in the Atlantic flyway wintered in the Guadalquivir marshes (including Doñana National Park) in southern Spain [28–29], but in recent years greylags have established new wintering areas, expanding their northern wintering range up to southern Sweden [30–32]. Thus, greylag geese wintering in western continental Europe are now spread over a latitudinal range of 2700 km. This geographical spread of the wintering area has been paralleled by a numerical increase across the flyway [29, 33].

Here, we analyze latitudinal changes in population trends and distribution of greylag geese wintering along the Atlantic flyway. We aim to identify the relative importance of land use changes and climate warming in explaining population increases during winter along the flyway. Given the recent expansion of wintering greylags towards the north, we predicted that population increase would be greater at northern than at southern wintering sites, not only due to warming that has increased the availability of winter food, but also because the traditional wintering sites further south would be closer to carrying capacity than “empty” northern sites. In addition, since the Guadalquivir marshes at the southern end of the flyway previously held most of the flyway population, and the timing of arrival of the geese has been recorded there for decades, we consider how the timing has changed over the years.

## Material and Methods

### Geese data

National totals for January count data from Sweden, Denmark, Germany, The Netherlands, Belgium and France during 1980–2009 were obtained from the International Waterbird Census (IWC, Wetlands International). Information from Spain during the same period was provided by the Monitoring Team of the Estación Biológica de Doñana (Guadalquivir marshes, which includes the Doñana National Park and surrounding areas), collected by the authors (Villafáfila, Nava, Boada and Pedraza lagoons, and Guadiana ricefields), or obtained from SEO/BirdLife (rest of Spain).

No specific permissions were required, as the study relies on census data collected during general surveys of wintering birds carried out in each location for other purposes, and not for the purpose of this paper. The study species is not endangered or protected, and no birds were collected or sampled, only counted from a distance.

We did not have access to count data at individual localities, except for Spain. We therefore used updated national maps with wintering distribution of greylag [34–40] to calculate the latitudinal centre of each national wintering population. Taking into account only the coordinates of the important wintering localities (3 major localities in Belgium and Spain, and localities with at least 250–1000 individuals in Sweden, 500–1500 in Denmark, 400–4000 in Germany, 5000 in The Netherlands, and 350–1450 in France) we took the average latitude between the most northern and the most southern localities for each country.

We used data from the literature [28] and personal observations from ornithologists and wardens of the Estación Biológica de Doñana to establish the date of first arrival of greylag geese to Doñana National Park in the Guadalquivir marshes in autumn every year since 1961. We did not include singletons, but arrival of the first flock of at least 5 individuals.

### Climate and land use data

**Fig 1:**
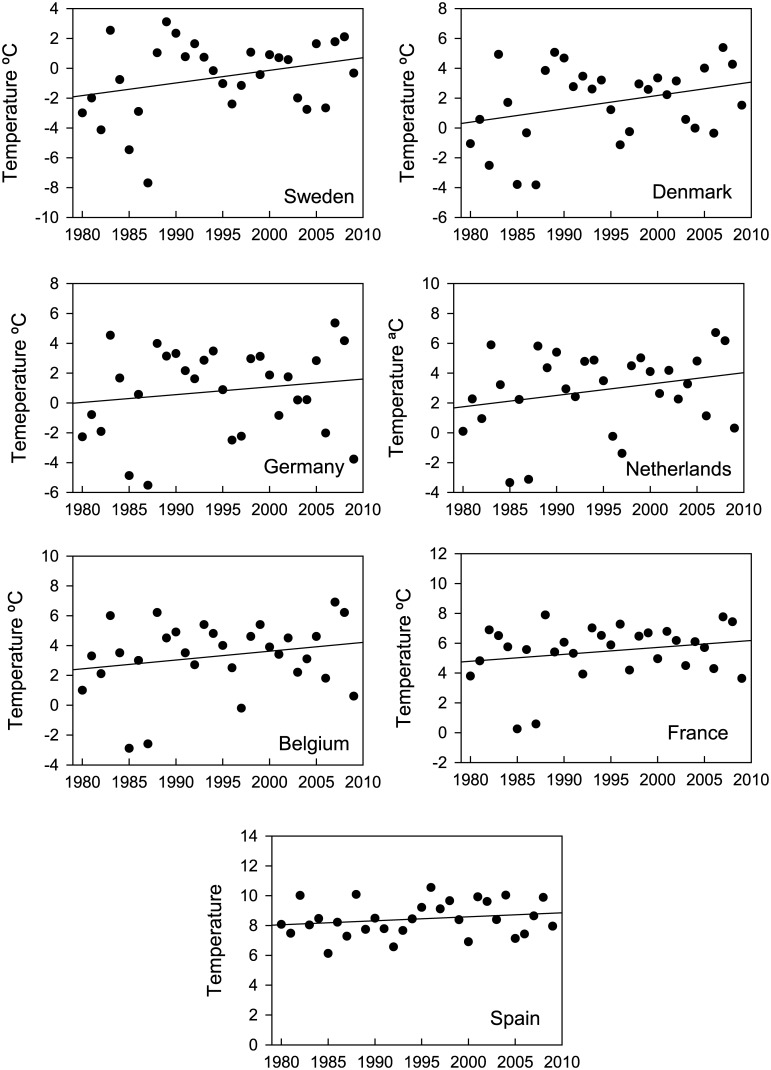
January mean temperatures in the wintering countries of greylag geese from 1980 to 2009, together with fitted linear regression lines.

As a measure of the variation in winter temperatures along the flyway we used the annual mean national temperature in January from 95 meteorological stations with complete datasets, located at altitudes below 700 m, and spanning the latitudinal range 36.5–58.4° N (http://www.cru.uea.ac.uk/data/, see S1 Table). According to linear regression there are positive, although not statistically significant, temperature trends in all countries, with increments ranging from 0.6°C in Spain, to 1.8°C in Denmark during 1980–2009 (Fig 1).

Agricultural land use data were extracted from Eurostat database statistics (http://epp.eurostat.ec.europa.eu/portal/page/portal/agriculture/agricultural_production/database, see S2 Fig). The main crops used by wintering geese were winter cereals (common winter wheat and winter barley), potatoes and sugar beet in Sweden [41], winter cereals and oilseed rape and sugar beet in Denmark [27], winter cereals and oilseed rape in Germany and France [27], winter cereals, potatoes and sugar beet in the Netherlands [42], winter cereals and potatoes in Belgium [38], and cereals and rice in Spain [43–45]. We therefore used the surface areas of these crops for further analyses (S2 Fig).

### Data analyses

Firstly, the TRIM (Trends and Indices for Monitoring Data) programme [46] was used to assess the long-term trends in winter populations in different countries. This software analyses time series of counts using Poisson regression, while correcting for any overdispersion and serial correlation in the data (see [46] for details). Due to the lack of IWC data for the early years in several countries, we only considered the period 1987–2009 so as to analyze trends in a comparable way.

Secondly, we performed linear regression models to determine the effects of winter temperature and crop surface areas on the number of wintering birds. In these models, the dependent variable was the annual census (log-transformed) in January, whereas year (as linear trend), mean temperature in January (°C) and surface areas (x1000 ha) of the different crops used by geese for each country were the predictors. All possible sub-models were generated from the general model using the MuMIn package in R (RCore Team 2014). We followed a model selection procedure based on Akaike’s Information Criteria (AIC; [47]). When several models differed in AIC by less than 2 we generated an averaged full model using MuMIn. Tests confirmed the normality and homoscedasticity of the residuals (only the model for France showed violation of these assumptions). We also performed an analysis of partial autocorrelation of the residuals from each model to determine if there was any temporal structure. No temporal autocorrelation was detected and hence we did not include any autoregressive terms in the models. To test relationships between pairs of variables, we used Pearson correlations. These analyses were performed using STATISTICA software (version 11; StatSoft, Tulsa, OK).

## Results

**Fig 2:**
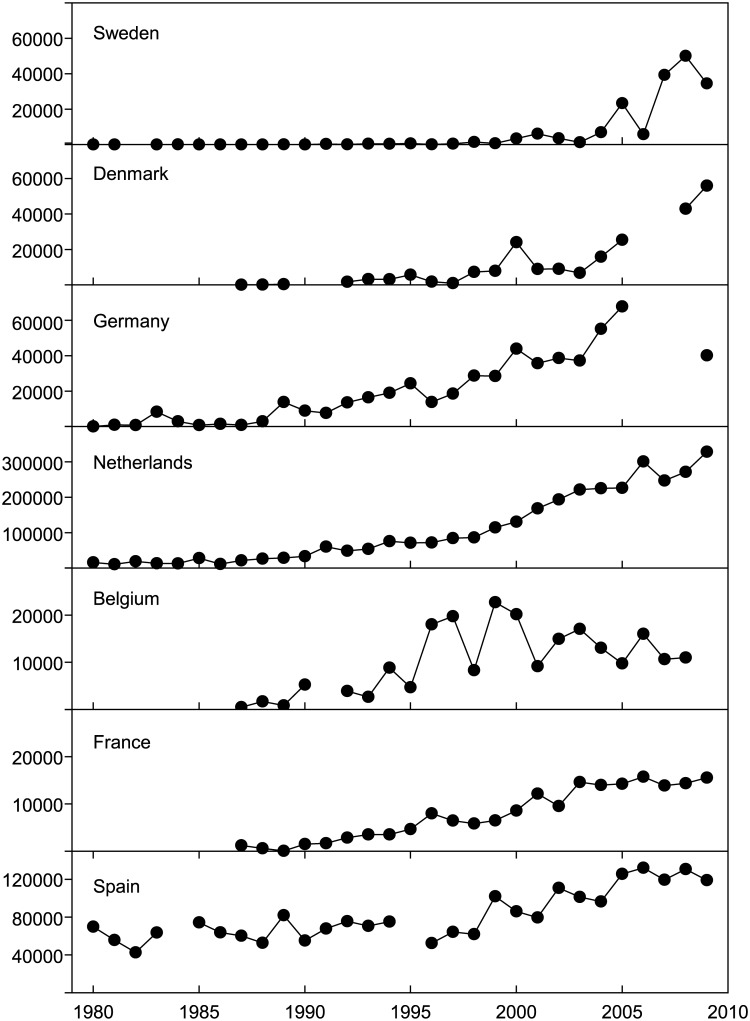
Winter greylag geese population estimates (mid January counts) in different countries of the Atlantic flyway between 1980 and 2009.

Numbers of wintering greylag geese have increased in all countries along the flyway during the last three decades (Fig 2). At the beginning of the 1980s, most geese wintered in Spain and to a much lesser extent in the Netherlands. Later, in the 1990s, the geese increased in numbers in France, Belgium, the Netherlands and Germany, and finally in the 2000s a similar pattern was registered in Denmark and Sweden. By 2009, the main wintering population was in the Netherlands (54% of the whole population), followed by Spain (20%), Denmark, Germany and Sweden (9, 7, and 6%, respectively), and France and Belgium (3 and 2%, respectively).

**Fig 3:**
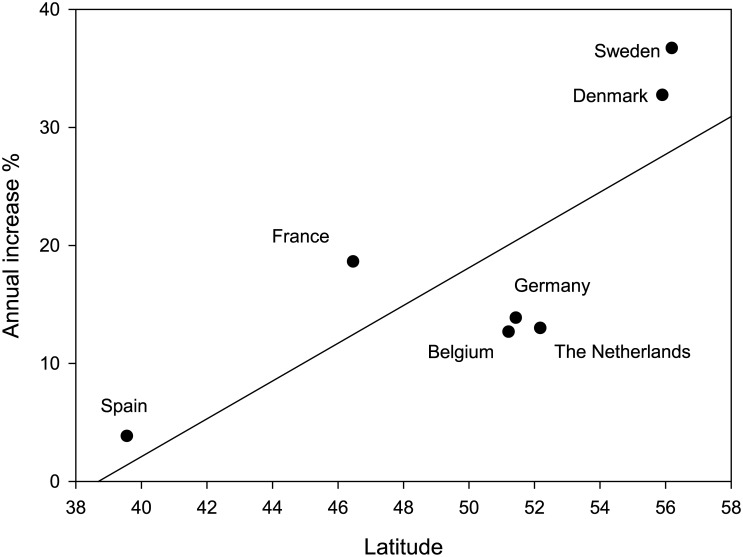
Annual increase during 1987–2009 of wintering greylag geese (see Table 1) in different countries along the Atlantic flyway in relation to latitude.

The annual increase in the number of wintering geese during 1987–2009 (Table 1) varied between 3.85% in the extreme south of the migratory route (Spain) and 36.73% in the North (Sweden), showing a significant positive relationship with latitude (r = 0.79; p = 0.04; Fig 3). While in most countries we did not observe any abrupt changes in the trends of wintering populations, the most northerly countries (Sweden and Denmark) experienced an abrupt point of inflection around the mid‒2000s, when rapid population increase began (Fig 2).

**Table 1:** Average and maximum winter population, multiplicative slopes and annual increase of greylag geese during winter in countries of the Atlantic flyway from 1987–2009 (1987–2008 for Belgium), estimated using the TRIM programme.

Country	Average	Maximum	MultiplicativeSlope	Std. error	Annual increase (%)
Norway	80	512	0.9915^ns^	0.0084	–0.85
Sweden	7810	50113	1.3673**	0.0043	36.73
Denmark	11043	55938	1.3276**	0.0067	32.76
Germany	25775	67741	1.1387**	0.0005	13.87
Netherlands	134387	328466	1.1300**	0.0002	13.00
Belgium	10429	22710	1.1268**	0.0009	12.68
France	7772	15738	1.1864**	0.0020	18.64
Spain	87313	132190	1.0385**	0.0001	3.85

(**, p<0.01; ns, non significant).

Results of regression models showed that the annual fluctuations in geese abundance were positively associated with the local temperature in January in Sweden, Denmark and Germany but negatively in Spain (Table 2). Indeed, a marked latitudinal trend in the effect of local temperature was apparent: from a negative value of the regression coefficient in the south to positive values in the north (with statistically significant effects in four countries). On the other hand, there was only one case in which land use changes were significantly associated with the number of wintering birds (the surface area occupied by winter cereals in Denmark). Finally, the wintering population size was positively and significantly associated with year in all countries (Table 2).

**Table 2:** Regression coefficients, adjusted standard error, values of t or z (for full averaged model) and p values from linear regression models between wintering greylag geese (log-transformed) as dependent variable and year, surface of crops and January temperatures as predictors. Only best sub-models are represented. When there is more than one sub-model with ΔAIC < 2 (see S2 Table) full model averaged coefficients are shown (see methods). Sample sizes vary greatly because of missing data for predictor variables, especially for land use.

		B	Adjusted SE	t/z	p
**Sweden**	Intercept	–598.48	37.85	15.81^1^	<0.001
n = 24 years	Year	0.30	0.02	16.14^1^	<0.001
	Temperature	0.18	0.05	3.35^1^	<0.001
	Sugar beet	-0.01	0.02	0.64^1^	0.525
**Denmark**	Intercept	–374.62	56.26	–6.66^2^	<0.001
n = 19 years	Year	0.19	0.03	6.63^2^	<0.001
	Winter cereals	0.01	0.00	4.93^2^	<0.001
	Temperature	0.39	0.06	6.21^2^	<0.001
**Germany**	Intercept	–282.40	71.75	3.94^1^	<0.001
n = 16 years	Year	0.15	0.04	4.04^1^	<0.001
	Temperature	0.11	0.04	3.10^1^	<0.01
	Oilseed rape	-0.00	0.00	0.93^1^	0.354
**Netherlands**	Intercept	–235.70	11.01	21.31^1^	<0.001
n = 30 years	Year	0.12	0.01	22.64^1^	<0.001
	Temperature	-0.01	0.01	0.49^1^	0.625
	Winter cereals	-0.00	0.00	0.57^1^	0.569
	Potaoes	0.00	0.00	0.37^1^	0.711
**Belgium**	Intercept	–528.65	88.25	–5.99^2^	<0.001
n = 12 years	Year	0.27	0.04	6.09^2^	<0.001
**France**	Intercept	–340.60	58.02	5.87^1^	<0.001
n = 23 years	Year	0.18	0.03	5.98^1^	<0.001
	Winter cereals	-0.00	0.00	0.77^1^	0.439
**Spain**	Intercept	–56.30	10.93	5.14^1^	<0.001
n = 28 years	Year	0.03	0.00	6.18^1^	<0.001
	Temperature	–0.09	0.03	3.47^1^	<0.001
	Rice	-0.00	0.00	0.71^1^	0.478

^1^z value;

^2^ t value

**Fig 4:**
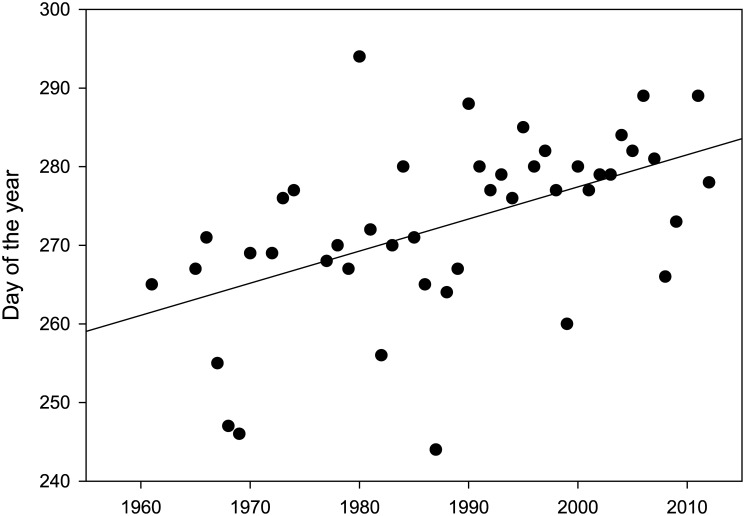
Trend in the first arrival of greylag geese to the Guadalquivir marshes (Doñana) in autumn between 1961 and 2012. The fitted regression line is: Day = − 539.954 + 0.4087 × Year (r = 0.516, P < 0.001).

We found a significant positive correlation between the date on which the first geese arrived to the Guadalquivir marshes in autumn, and year (y = −539.95 + 0.41 x; r = 0.52; p < 0.001; Fig 4). In the 1960s, the first arrivals took place in late September, but over the years they have gradually become later, with an estimated delay of 4 days per decade.

## Discussion

Winter populations of greylags have increased during the last decades in all countries along the Atlantic flyway. The high quality of the census data has allowed us to demonstrate clear spatial and temporal patterns. The further north the wintering area: 1) the faster the increase has been, and 2) the later this increase has occurred. Furthermore, our regression models revealed that the response of wintering populations to changes in temperature switches from being positive in the north of the flyway to negative in the southern extreme.

Lehikoinen et al. [18] found that a shift in the wintering distributions of three duck species in Europe correlated with an increase in winter temperature in the north-eastern part of the wintering area, where bird abundance increased, corresponding with decreases in abundance at south-western sites. In our case, the greylag goose populations are still increasing in all countries, although there have been a northward expansion and a change in the centre of gravity: in the 1980s, Spain hosted almost all wintering geese, while in 2009 the bulk of the population was in The Netherlands, and 15% of geese wintered further north in Sweden and Denmark.

On the other hand, the later arrival recorded over time in the Guadalquivir marshes is entirely consistent with changes in greylag migration reported for other countries (see S4 Table). During the last decades, the geese arrived earlier to the breeding grounds and spent more time in northern areas, delaying their arrival to southern wintering grounds. This pattern is consistent with an effect of climate change. When compared with the increases in temperature over time (Fig 1), the changes in migration phenology over the same period show stronger and more significant patterns, suggesting that geese can advance their phenology to keep track with, or faster than, climate change. Voslamber et al. [35] already suggested that climate change explains why greylags breeding in the Netherlands have reduced their tendency to migrate south over the last 20 years.

Climate warming does not have the same effect on winter conditions along the flyway. In Spain, France, Belgium and the Netherlands winter temperatures (average around 8.4, 5.5, 3.3, and 3.2°C in January during 1980–2009, respectively) are not usually a limiting factor for geese, but in Sweden, Denmark and Germany mean temperatures in January usually fall below 0°C (Fig 1), limiting food availability as foraging habitats freeze. In recent decades, northern countries have experienced a greater increase in temperature [1]. In southern Sweden, the proportions of nights and days that fell below 0°C in winter showed a substantial decrease of 5–10% and 5‒15%, respectively, from 1950 to 2011 [48]. Thus, warming can increase the access to feeding resources in northern sites. In Sweden, very few greylags were found in the country in January before the late 1990s, but in more recent mild years up to 25% of the September totals remained in the country for the winter [32]. In addition, in milder winters the arrival of the first geese to the breeding areas from the wintering grounds may advance, increasing the winter population in these areas [30].

It could be argued that the increasing population of geese in non-traditional wintering areas might be due to a ‘buffer effect’, which occurs when migratory individuals occupy the best habitat areas first and later they spread to poorer sites during a period of population growth [5]. This buffer effect has been demonstrated in the increasing population of another long-distance migratory waterbird, the black-tailed godwit *Limosa limosa islandica* [49–50]. However, we can discard a buffer effect as a major density-dependent process acting on the greylag population, because the newly occupied areas seem to be of higher quality than traditional areas. Shifts in the wintering location of individually marked geese from Spain to the Netherlands have been recorded [12, 30], and greylag geese breeding in Scania (South of Sweden) and wintering in non-traditional areas (the Netherlands) not only arrived earlier, but also had better survival rates and reproductive success than those wintering in Spain [30, 51–52]. In other words, the latitudinal effects we have recorded seem to be a consequence of a combination of three factors: individual geese changing their choice of wintering sites; individuals wintering further north having higher survival, and individuals wintering further north having higher reproductive success, contrary to the buffer effect.

Although the energetic benefits of migrating longer rather than shorter distances have been demonstrated in black-tailed godwit [9–10], data on European spoonbills *Platalea leucorodia* suggests that flying further does not necessarily yield fitness benefits [53–54]. The higher reproductive success and lower mortality of geese wintering further north could be due to the lower direct costs of migration, or alternatively could reflect a difference in individual quality between birds choosing to winter in the south and those staying further north.

Changes in land use have also been important along the Atlantic flyway. Agricultural practices such as expansion of oilseed rape, winter cereals, sugar beet, potatoes and nitrogen inputs to grasslands, have enhanced the carrying capacity of winter habitats for greylags [32, 41, 55]. Nowadays, wintering geese rely on food resources offered by agricultural fields, which represent about 70% of the land surface area in the Netherlands [42], where there is a positive correlation between the degree of agricultural exploitation by greylag geese and its population size [55]. Nevertheless, the area dedicated to these crops only experienced important increases since 1980 in one country, Denmark (S2 Fig). A significant partial effect of the surface area of winter cereals on the wintering numbers of geese (Table 2) indicates that changes in land use can only be considered to have had a major role in explaining the increase in goose numbers in Denmark. A similar situation has occurred with the pink-footed goose (*Anser brachyrhynchus*), as the increase in the winter population of this species in Denmark coincided with the increase in surface area of winter cereals [24]. Increases in the quality of agricultural habitat may be important as well as quantity, but unfortunately we had no suitable measure of quality for our analyses.

Because greylag geese are a quarry species, hunting mortality may contribute to the costs of migration, and changes in hunting pressure could possibly contribute to the general population increase, and to the changes in population trend with latitude. However, available data do not support a role for hunting mortality, as there is no evidence that this has decreased in Europe. During the 1970s, the total hunting bag of this flyway population was estimated at 10,000, which represented around 30% of the whole population [56]. More than three decades later, an estimated 107,813 geese were shot annually (30.8% of the winter population, [57]). In the Netherlands alone, 80,793 and 132,720 geese were shot under management schemes or with special permits in the 2007/2008 and 2010/2011 seasons respectively (30% and 29.2% of the January Netherlands population, [58–59]). Furthermore, there has been a reduction in hunting pressure in the Guadalquivir marshes owing to an extension of protected areas and a reduction in the number of days when hunting is permitted [60]. Nevertheless this has not led to an increase in the numbers of wintering greylags [61]. Clearly, it is unlikely that the relationship between population trend and latitude can be explained on the basis of hunting.

Our regression models that attempt to account for the effects of climate warming and the changes in land-use do not fully explain the winter population trends, as indicated by our result that the partial effect of year remains significant in the models for all countries. These results may partly be because the predictor variables we used do not fully represent the complexities of changes in land use (e.g. the changes in practice within a given crop type) or climate change (e.g. changes in wind speed or other parameters influencing the thermal biology of geese). The high intrinsic growth rates in the wintering populations in a given area are also likely to be related to global changes in other areas along the flyway, especially in breeding sites. For example, a general reduction in adult mortality with time across the flyway could contribute to the strong, universal year effect.

Apart from impacts on agriculture, which in the Netherlands constitutes an important problem [42], the major expansion in the total number of greylags in this flyway population may have negative consequences for conservation of natural habitats in the breeding areas, now used also as wintering areas, as observed for other expanding geese species. In North America, increasing numbers of snow geese (*Chen caerulescens*) have led to loss of vegetation, and exposure and partial erosion of sediment, resulting in the loss of intertidal saltmarshes and the establishment of an alternative stable state (exposed sediments) [62]. In Dutch wetlands, grazing by greylags in combination with other herbivorous waterbirds is already reducing the species richness and diversity of riparian vegetation [63]. Furthermore, in Belgium and the Netherlands, greylags and alien Canada geese *Branta canadensis* are already causing similar conflicts by degrading parks and urban wetlands [64]. Potential impacts of greylags may also be exacerbated by the changing migration phenology, since the geese are spending successively more days a year in the breeding areas.

In conclusion, climate warming may have facilitated latitudinal-related increases in wintering populations of greylag geese by enhancing the carrying capacity of habitats at northern latitudes. Local temperature effects detected in our models are consistent with a causal effect of climate change, since the population increase is related to changes in temperature. Our findings may allow the formulation of predictions for long term consequences on the size of wintering populations in different sites. Thus, as temperatures continue to increase during this century [1], it is expected that the trend that we have documented here will be exacerbated, which may lead to a decline in the number of greylag geese wintering in historical southern sites and further northward expansion of the wintering range. Recent censuses in the main wintering localities in Spain (which hold 90% of the geese in Spain) are in line with this prediction, showing a 15% decrease in mean geese numbers (from 100,225 birds in 2000–2009 to 85,141 in 2010–2013). The change in migration phenology at the southern end of the flyway itself suggests that the southernmost limit of the wintering range will begin to contract within the coming decades.

## Supporting Information

S1 Fig Surface area of crops. (PDF)

S1 Table Meteorological stations considered in this study. (PDF)

S2 Table Models. (PDF)

S3 Table Changes reported in the timing of graylag geese migration in the Atlantic flyway. (PDF)
