# Supplementary material for: Latitudinal-Related Variation in Wintering Population Trends of Greylag Geese (Anser Anser) along the Atlantic Flyway: A Response to Climate Change?
Source: PLoS One. 2015 Oct 14;10(10):e0140181. doi: 10.1371/journal.pone.0140181 (PMC4605798; doi:10.1371/journal.pone.0140181)
Supplement: S1 Fig — (PDF) [file pone.0140181.s001.pdf]

S1 Fig. Surface of crops. Data from Eurostat (http://epp.eurostat.ec.europa.eu/portal/page/portal/agriculture/agricultural_production/database).

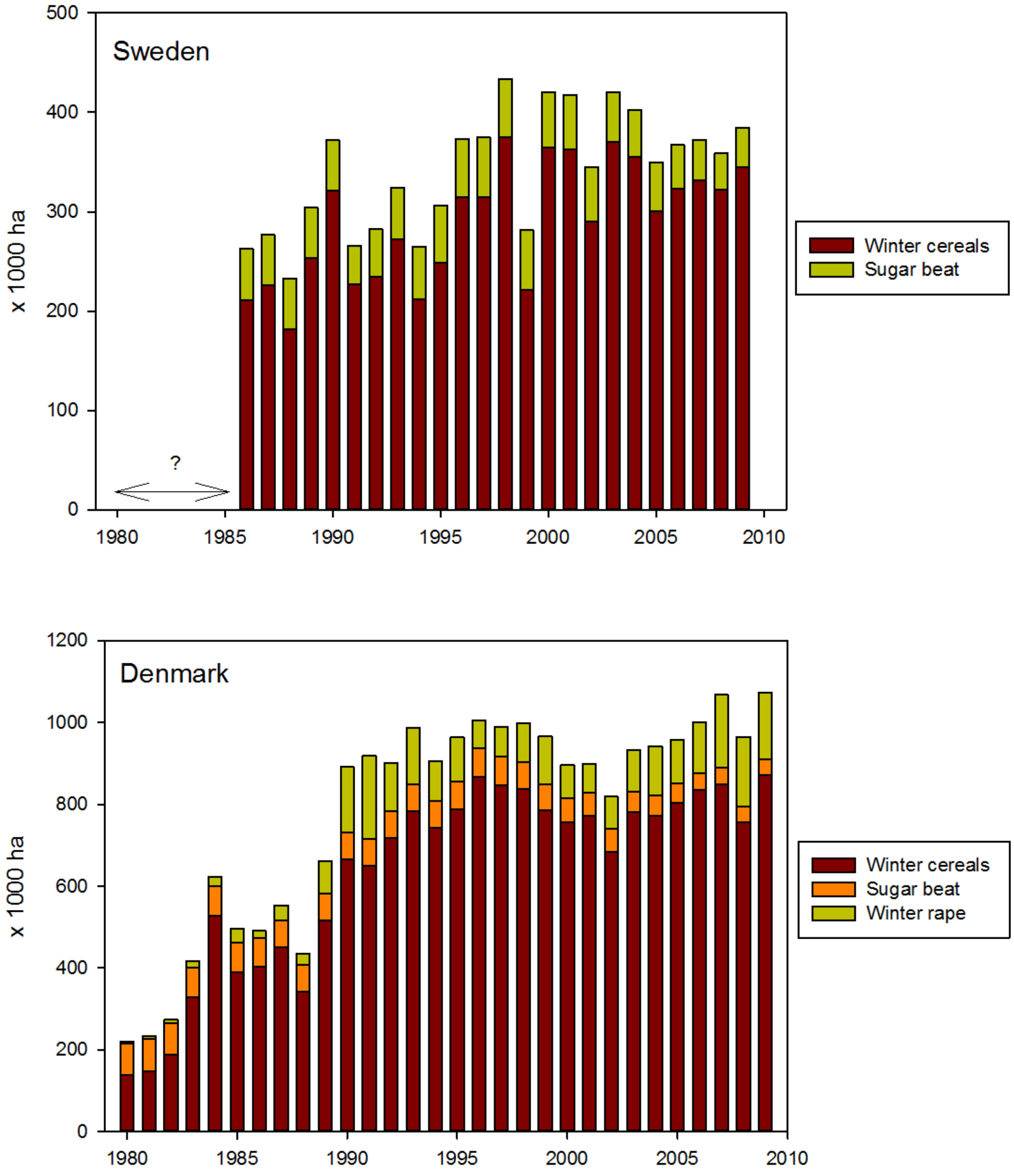

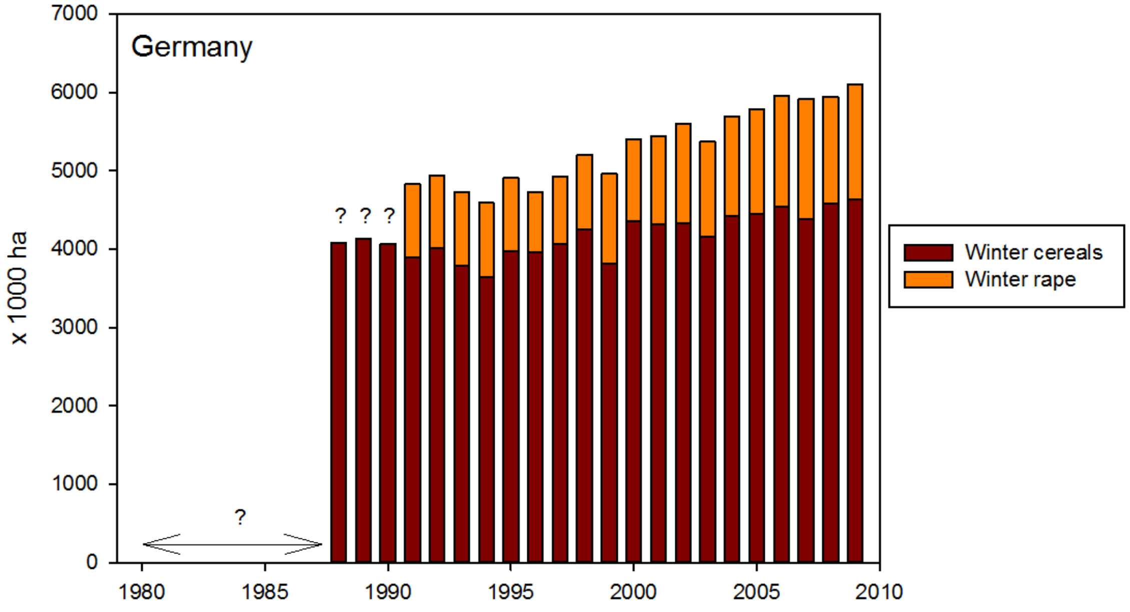
Germany
x 1000 ha
7000
6000
5000
4000
3000
2000
1000
0
1980
1985
1990
1995
2000
2005
2010
? ? ?
?
Winter cereals
Winter rape

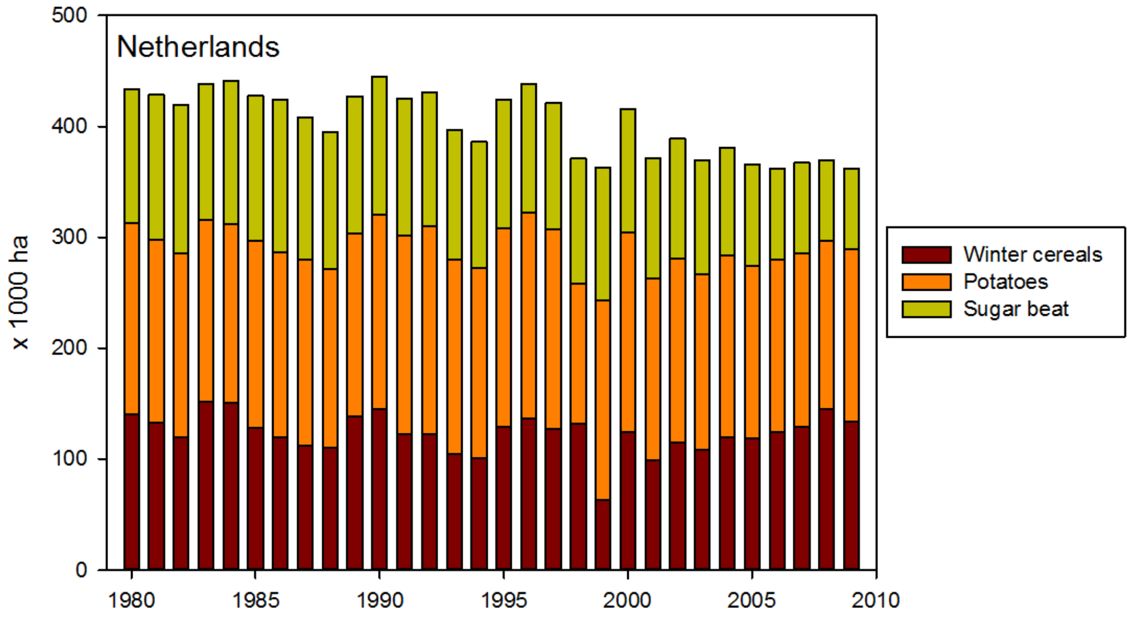
Netherlands
x 1000 ha
500
400
300
200
100
0
1980
1985
1990
1995
2000
2005
2010
Winter cereals
Potatoes
Sugar beat

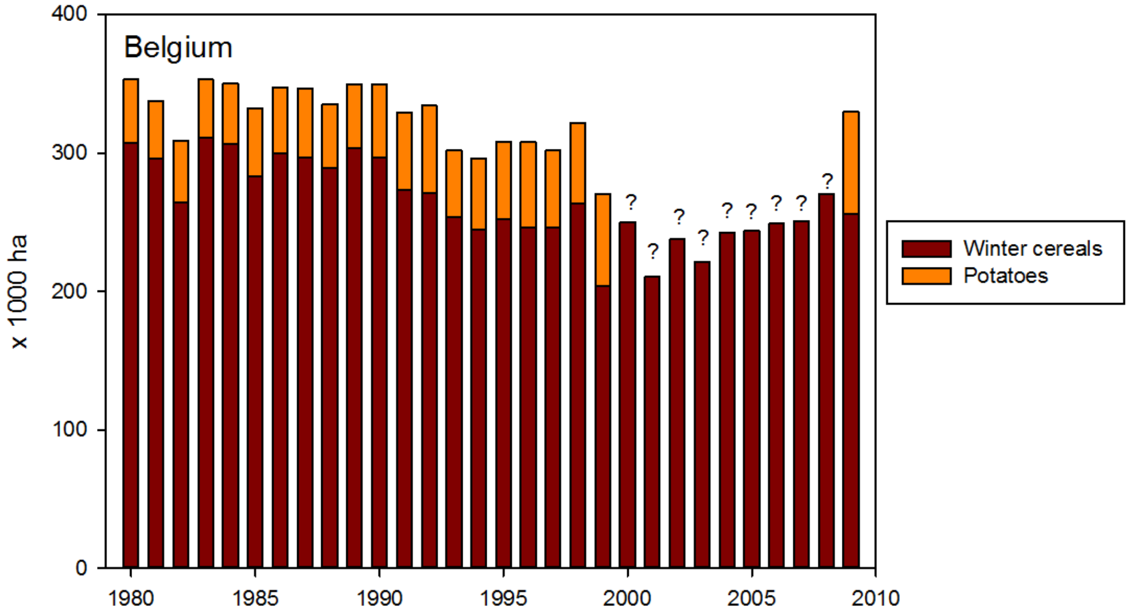
Belgium
x 1000 ha
400
300
200
100
0
1980
1985
1990
1995
2000
2005
2010
?
Winter cereals
Potatoes

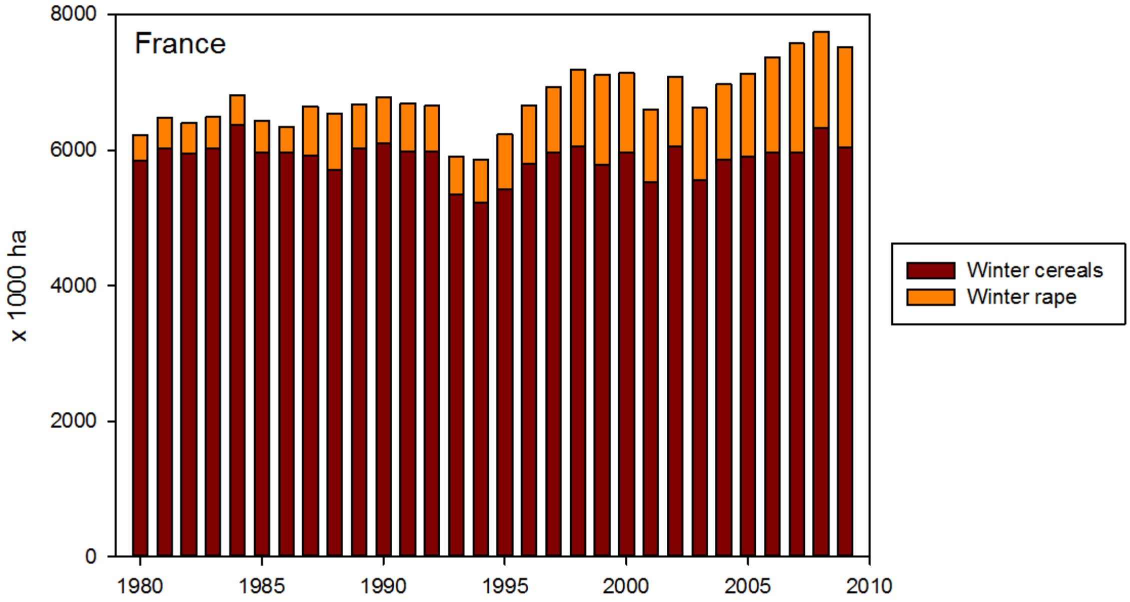
France
x 1000 ha
8000
6000
4000
2000
0
1980
1985
1990
1995
2000
2005
2010
Winter cereals
Winter rape

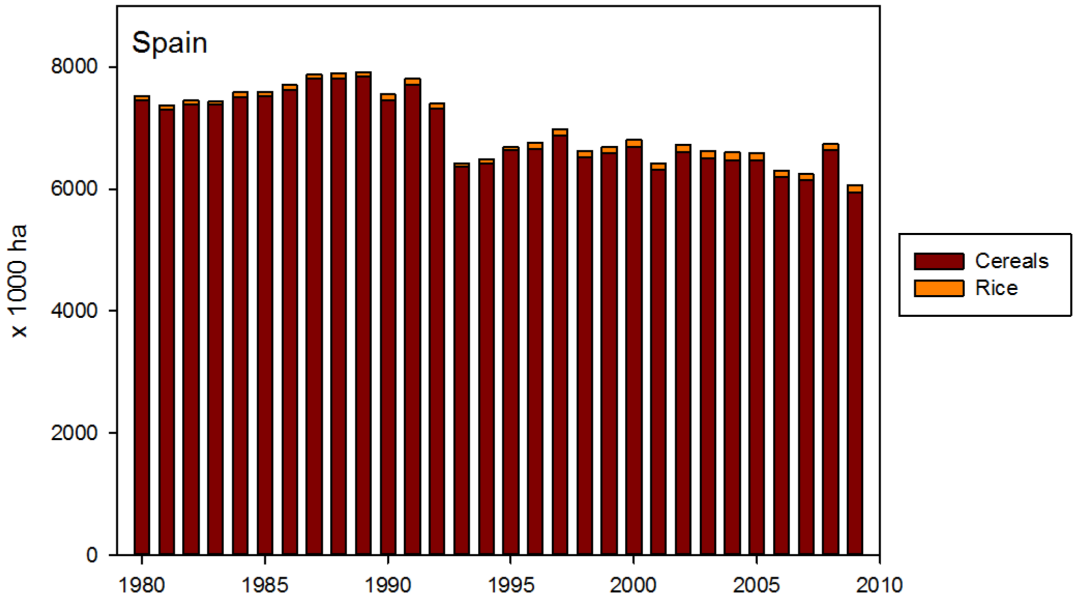
Spain
x 1000 ha
0
2000
4000
6000
8000
1980
1985
1990
1995
2000
2005
2010
Cereals
Rice
