# Supplementary material for: Latitudinal-Related Variation in Wintering Population Trends of Greylag Geese (Anser Anser) along the Atlantic Flyway: A Response to Climate Change?
Source: PLoS One. 2015 Oct 14;10(10):e0140181. doi: 10.1371/journal.pone.0140181 (PMC4605798; doi:10.1371/journal.pone.0140181)
Supplement: S1 Table — (PDF) [file pone.0140181.s002.pdf]

S1 Table. Meteorological stations considered in this study. Data from http://www.cru.uea.ac.uk/data/

Number	Name	Country	Latitude	Longitude	Height
25500	Jonkoping Flygplats	Sweden	57,8	14,1	226
25890	Gotska Sandoen	Sweden	58,4	19,2	12
25900	Visby	Sweden	57,7	18,3	42
60510	Vestervig	Denmark	56,8	8,3	18
60880	Nordby	Denmark	55,4	8,4	5
61320	Tranebjerg	Denmark	55,9	10,6	11
61860	Koebenhavn	Denmark	55,7	12,5	9
61930	Hammerodde-Fyr	Denmark	55,3	14,8	11
62400	Amsterdam (Schipol)	Netherlands	52,3	4,8	-4
62600	De Bilt	Netherlands	52,1	5,2	2
62750	Deelen	Netherlands	52,1	5,9	50
62800	Eelde	Netherlands	53,1	6,6	4
62830	Winterswijk/Hupsel	Netherlands	52,1	6,7	29
63440	Rotterdam	Netherlands	52,0	4,5	-5
63500	Oudenbocsh/Gilze-Riz	Netherlands	51,6	4,9	11
63700	Eindhoven	Netherlands	51,5	5,4	22
63750	Gemert/Volkel	Netherlands	51,7	5,7	20
63800	Maastricht	Netherlands	50,9	5,8	114
64470	Brussels/Uccle/Cbt	Belgium	50,8	4,4	100
71100	Brest/Guipavas	France	48,5	-4,4	98
71900	Strasbourg Entzhei	France	48,6	7,6	150
72220	Nantes	France	47,2	-1,6	26
72550	Bourges	France	47,1	2,4	161
72800	Dijon-Longvic Airpor	France	47,3	5,1	227
74340	Limoges/Bellegarde	France	45,9	1,2	402
75100	Bordeaux/Merignac	France	44,8	-0,7	47
76300	Toulouse/Blagnac	France	43,6	1,4	151
76500	Marseille Marignag	France	43,4	5,2	5
76900	Nice Airport	France	43,7	7,2	4
77470	Perpignan	France	42,7	2,9	42

80010	La Coruña	Spain	43,4	-8,4	67
80230	Santander	Spain	43,5	-3,8	64
80270	San Sebastián	Spain	43,3	-2,0	252
80850	Pamplona	Spain	42,8	-1,6	452
80940	Huesca	Spain	42,1	-0,3	541
81410	Valladolid	Spain	41,6	-4,7	691
81600	Zaragoza	Spain	41,7	-1,0	245
81800	Barcelona	Spain	41,4	2,2	420
82210	Madrid/Barajas	Spain	40,5	-3,6	609
82800	Albacete	Spain	39,0	-1,9	699
82850	Valencia	Spain	39,5	-0,4	11
83300	Badajoz	Spain	38,9	-6,8	185
83480	Ciudad Real	Spain	39,0	-3,9	627
83590	Alicante	Spain	38,4	-0,5	82
83830	Huelva	Spain	37,3	-6,9	19
83900	Sevilla	Spain	37,4	-5,9	31
84200	Granada	Spain	37,1	-3,6	685
84300	Murcia	Spain	38,0	-1,1	57
84530	Cadiz-San Fernando	Spain	36,5	-6,2	30
84820	Malaga	Spain	36,7	-4,5	7
84870	Almería/Aeropuerto	Spain	36,9	-2,4	15
90916	Putbus	Germany	54,7	13,4	42
100150	Helgoland	Germany	54,2	7,9	4
100200	List_Auf_Sylt	Germany	55,0	8,4	26
100350	Schleswig	Germany	54,5	9,6	43
101130	Norderney	Germany	53,7	7,2	11
101310	Cuxhaven	Germany	53,9	8,7	5
101470	Hamburg-Fuhlsbuettel	Germany	53,6	10,0	16
101620	Schwerin	Germany	53,6	11,4	59
101700	Warnemunde	Germany	54,2	12,1	4
101840	Griefswald/Wieck	Germany	54,1	13,5	1
102240	Bremen	Germany	53,1	8,8	4
102700	Neuruppin	Germany	52,9	12,8	38

102910	Angermuende	Germany	53,0	14,0	54
103380	Hannover	Germany	52,5	9,7	55
103610	Magdeburg	Germany	52,1	11,6	85
103790	Potsdam	Germany	52,4	13,1	81
103820	Berlin-Tegel_Airport	Germany	52,6	13,3	36
103840	Berlin	Germany	52,5	13,4	50
103930	Lindenberg	Germany	52,2	14,1	98
104000	Duesseldorf_Airport	Germany	51,3	6,8	37
104100	Essen	Germany	51,4	7,0	152
104300	Lippspringe,_Bad	Germany	51,8	8,8	157
104690	Leipzig	Germany	51,3	12,4	148
104880	Dresden-Klotz	Germany	51,1	13,8	232
104960	Cottbus	Germany	51,8	14,3	69
104990	Gorlitz	Germany	51,2	15,0	237
105010	Aachen	Germany	50,8	6,1	177
105130	Koeln-Bonn_Airport	Germany	50,9	7,2	92
105320	Giessen/Wettenberg	Germany	50,6	8,7	203
105480	Meiningen	Germany	50,6	10,4	450
105540	Erfurt/Bindersleben	Germany	51,0	11,0	314
105670	Gera-Leumnitz	Germany	50,9	12,1	311
106090	Trier-Petrisberg	Germany	49,7	6,7	265
106370	Frankfurt/m.	Germany	50,1	8,6	113
106550	Wuerzburg	Germany	49,8	10,0	268
106750	Bamberg	Germany	49,9	10,9	240
106850	Hof	Germany	50,3	11,9	565
106880	Weiden	Germany	49,7	12,2	440
107080	Saarbruecken	Germany	49,2	7,0	191
107290	Mannheim	Germany	49,5	8,6	96
107380	Stuttgart/Echterd	Germany	48,7	9,2	396
107420	Oehringen	Germany	49,2	9,5	276
107630	Nuernberg_Airport	Germany	49,5	11,1	314
109290	Konstanz	Germany	47,7	9,2	443
