# Supplementary material for: Latitudinal-Related Variation in Wintering Population Trends of Greylag Geese (Anser Anser) along the Atlantic Flyway: A Response to Climate Change?
Source: PLoS One. 2015 Oct 14;10(10):e0140181. doi: 10.1371/journal.pone.0140181 (PMC4605798; doi:10.1371/journal.pone.0140181)
Supplement: S2 Table — (PDF) [file pone.0140181.s003.pdf]

S2 Table. Models

Country	Model	AICc	ΔAICc	Weight
Sweden	year + temperature	48.2	0.00	0.572
	year + temperature + sugar beat	48.8	0.58	0.428
Denmark	year+ temperature + cereals	42.6	0.00	0.499
Germany	year + temperature + rape	5.38	0.00	0.59
	year + temperature	6.10	0.72	0.41
The Netherlands	year	3.4	0.00	0.266
	year + cereals	3.7	0.32	0.227
	year + potatoes	4.1	0.66	0.191
	year + temperature	4.3	0.83	0.176
	year + temperature + cereals	4.7	1.29	0.140
Belgium	year	28.2	0.00	0.687
France	year + cereals	64.2	0.00	0.52
	year	64.4	0.16	0.48
Spain	year + temperature	-19.7	0.00	0.52
	Year + temperature + rice	-19.6	0.16	0.48
