# Supplementary material for: Latitudinal-Related Variation in Wintering Population Trends of Greylag Geese (Anser Anser) along the Atlantic Flyway: A Response to Climate Change?
Source: PLoS One. 2015 Oct 14;10(10):e0140181. doi: 10.1371/journal.pone.0140181 (PMC4605798; doi:10.1371/journal.pone.0140181)
Supplement: S3 Table — (PDF) [file pone.0140181.s004.pdf]

S3 Table. Changes reported in the timing of greylag geese migration in the Atlantic flyway.

Locality	Migration	Change in dates	Period	Source
Western France (Passage)	Spring	From early February to mid-January	1980-2005	[14]
Vega (Norway) (Breeding)	Spring	5-7 days earlier per decade	1971-2004	[13]
Scania (Sweden) (Breeding)	Spring	20 days earlier	1986-2002	[12]
Netherlands	Autumn	40 days later (from Scania)	1986-2002	[12]
Guadalquivir marshes (Spain)	Autumn	4 days later per decade	1961-2012	This paper
